# Optimized Preparation of *Gastrodiae elata* Extract Enhances Antiepileptic Effects by Regulating Neuroinflammation, Oxidative Stress, and Neuronal Apoptosis in Rats

**DOI:** 10.3390/cimb48070688

**Published:** 2026-07-03

**Authors:** He Wang, Shiyi Lun, Hu Ding, Zhimeng Li, Xian Wu, Huiyang Yuan, Bo Yang, Guoxin Ji, Huan Wang, Shumin Wang

**Affiliations:** 1 College of Pharmacy, Changchun University of Chinese Medicine, Changchun 130117, China; 15044100183@163.com (H.W.); 15662248398@163.com (S.L.); lzm2084@163.com (Z.L.); 15043926050@163.com (X.W.); 15104468789@163.com (H.Y.); yangbo0623@126.com (B.Y.); 17843099892@163.com (G.J.); 2 Jilin Ginseng Academy, Changchun University of Chinese Medicine, Changchun 130117, China; dh1291728695@163.com

**Keywords:** *Gastrodia elata* Bl., yellow rice wine processing, epilepsy, neuroinflammation, active components, antioxidant activity

## Abstract

Epilepsy is a common chronic neurological disorder characterized by recurrent seizures. *Gastrodia elata*, the dried tuber of *G. elata* Bl. (Orchidaceae), is a valuable medicinal and edible botanical resource. This study optimized the preparation of Yellow Rice Wine-Processed *G. elata* (YPGE) and investigated its antiepileptic effects and underlying mechanisms in a pentylenetetrazol (PTZ)-kindled rat model. Processing parameters were optimized using single-factor experiments combined with an analytic hierarchy process (AHP)-entropy weight method (EWM) weighting strategy and Box–Behnken design–response surface methodology. The optimal parameters were determined as 18% alcohol by volume, 72 °C drying temperature, and 32 h drying time. Compared with unprocessed *G. elata* (GE), YPGE exhibited 0.54-, 0.13-, 1.87-, and 3.58-fold increases in the contents of gastrodin (GAS), *G. elata* polysaccharides (GEPs), p-hydroxybenzyl alcohol (*p*-HBA), and total parishins (TP), respectively, and demonstrated significantly enhanced in vitro antioxidant activity (IC50 values of 2.604, 2.719, and 4.046 mg/mL for DPPH, ABTS, and hydroxyl radicals). In vivo, both GE and YPGE significantly reduced seizure severity, decreased inflammatory cytokines (TNF-α, IL-1β), alleviated oxidative stress (increased SOD and GSH-Px, decreased MDA), and modulated neurotransmitter balance (reduced Glu, increased GABA) in brain tissues. YPGE also upregulated P-glycoprotein expression and reduced neuronal apoptosis in the hippocampal CA1 region by upregulating Bcl-2 and downregulating Bax. These findings suggest that YPGE exerts multi-target antiepileptic effects through synergistic anti-inflammatory, antioxidant, and anti-apoptotic actions, providing experimental evidence for the development of novel antiepileptic therapies based on processed *G. elata*.

## 1. Introduction

Epilepsy is a chronic neurological disorder caused by abnormal electrical discharges of brain neurons. Due to its complex pathogenesis, high incidence and mortality rates, it has brought many negative impacts on people’s quality of life. Epileptic seizures affect approximately 65 million people worldwide and about 1% of the U.S. population, with higher incidence rates among those under 1 year old and over 85 years old [1]. Therefore, the prevention and improvement of epilepsy as well as the research on its pathogenesis have long been the focus of research in various fields. Modern studies have shown that currently commonly used [2] antiepileptic drugs such as carbamazepine, gabapentin, and pregabalin can quickly reach the whole body to take effect but may cause side effects such as nausea, vomiting, dizziness, drowsiness, diplopia, vertigo, and ataxi [3,4,5]. During pregnancy, taking antiepileptic drugs may increase the incidence of childhood mental disorders such as autism and epilepsy [6,7,8,9].

Modern medical research has indicated that the occurrence of neurodegenerative diseases, cancer, cardiovascular diseases, retinopathy, and skin diseases is closely associated with oxidative stress [10,11,12,13]. In modern pharmacological studies related to epilepsy, inflammation can exacerbate blood–brain barrier disruption, neuronal damage, and glial cell activation, forming an “epilepsy-inflammation” vicious cycle; during epileptic seizures, high-frequency neuronal firing leads to abnormalities in the mitochondrial electron transport chain, generating superoxide anions (O_2_^−^), hydrogen peroxide (H_2_O_2_), etc. Activated microglia release reactive oxygen species (ROS) through NADPH oxidase, exacerbating local oxidative damage. To prevent the occurrence of oxidative stress, the human body can maintain adequate levels of antioxidants by ingesting foods rich in antioxidants [14]. Therefore, natural antioxidants are of great significance for the scavenging of free radicals.

During the pathological progression of epilepsy, the expression of pro-apoptotic proteins (Bax, Bak) is up-regulated, while the expression of anti-apoptotic proteins (Bcl-2, Bcl-xL) is down-regulated. The imbalance of the Bax/Bcl-2 ratio is a core hallmark of apoptosis. Wang L [9] and others established a pentylenetetrazol (PTZ)-kindled chronic epilepsy model in mice and conducted research on related inflammation; it was found that semaglutide could reduce the release of inflammatory factors IL-1β, IL-18, IL-6, and TNF-α (*p* < 0.01 or *p* < 0.001), as it significantly upregulates the protein expression of NLRP3, ASC and caspase-1 p20 in BV2 cells. Semaglutide pretreatment could also inhibit its activation (*p* < 0.05). Nissl staining showed that semaglutide can increase the number of neurons in the hippocampal CA1/CA3 regions and reduce nuclear fragmentation [15]. Xie R [16] and others studied the neuroprotective effect and mechanism of quercetin on epilepsy. Quercetin exerts synergistic effects through multiple pathways, including inhibiting inflammatory responses, enhancing antioxidant capacity, and suppressing cell apoptosis, reducing neuronal damage and cognitive dysfunction caused by epileptic seizures. The mechanism of action mainly relies on the activation of the Nrf2/SIRT1 pathway as well as the regulation of inflammatory factors, oxidative products, and apoptosis-related proteins.

*G. elata* is a perennial symbiotic herb belonging to the family Orchidaceae. As a valuable traditional Chinese medicinal material, *G. elata* has attracted considerable attention owing to its active constituents, including GAS, *G. elata* polysaccharides (GEPs), and parishins, which have been demonstrated to exhibit potent antioxidant activities [17,18]. Furthermore, *G. elata* exemplifies the TCM concept of “medicine–food homology” and has been widely used in dietary therapy and medicinal practice across Asia for thousands of years [19]. Geographically, *G. elata* is primarily distributed in Chinese provinces, including Yunnan, Guizhou, Sichuan, Tibet, and Shaanxi, as shown in Figure 1, and is also found in Japan, India, and Nepal, constituting a broad natural habitat that supports its sustainable use as both a medicinal and edible resource [20]. In traditional Chinese medicine, it is renowned for its remarkable effects in calming endogenous wind to relieve spasm, suppressing liver yang, and dispelling wind to dredge collaterals. As a traditionally important medicinal herb for treating conditions such as vertigo, headache, and limb numbness, its medicinal value has been fully validated in clinical practices throughout history [21,22,23]. Modern studies have further revealed that *G. elata* is rich in various bioactive components, including polysaccharides, polyphenols, amino acids, and glycosides. These components not only form the material basis for its medicinal effects but also endow it with multiple properties such as antioxidant and neuroprotective activities [24,25,26,27,28]. With increasing market recognition, the application scope of *G. elata* in the food sector has expanded, and its usage continues to grow. The synergistic release of its dual medicinal and edible values is demonstrating an increasingly broad comprehensive market prospect [29]. In China’s traditional processing system, *G. elata* processing has mainly formed two technical routes: one is basic processing such as steaming, boiling and stir-frying; the other is a composite processing method combined with auxiliary materials like yellow rice wine and ginger. Both can specifically improve the flavor and ensure subsequent edible value [20,30,31]. Taking steaming as an example, studies by Li et al. show that the contents of active components such as gastrodin (GAS), p-hydroxybenzyl alcohol (*p*-HBA), parishin B (PB) and parishin C (PC) in *G. elata* continue to increase during the steaming process, which confirms the promoting effect of scientific processing on improving component conversion efficiency [32]. Ancient medical classics clearly record that processing *G. elata* with yellow rice wine can enhance the efficacy of YPGE in dispelling wind, dredging collaterals, calming the liver and extinguishing wind, and at the same time make its texture easier to integrate into dietary forms such as soups and medicinal diets [33,34]. Therefore, optimizing the processing technology of YPGE has urgent practical significance. It can not only improve the quality of this processed product and enhance its dual medicinal and edible value but also provide a scientific reference for its standardized application in production, clinical practice and food fields.

Based on the above background, for the first time, this study integrates subjective and objective weighting methods using the analytic hierarchy process (AHP) and entropy weight method (EWM), to construct a comprehensive evaluation index system. Combined with the Box–Behnken design–response surface method (BBD-RSM), it systematically optimizes the processing parameters of YPGE. After determining the optimal processing parameters, the contents of active components and in vitro antioxidant activities of *G. elata* before and after processing were compared. Meanwhile, an exploratory study was conducted on the antiepileptic pharmacological effects of YPGE using various experimental methods, including the Morris water maze, HE staining, TUNEL staining, ELISA, and Western Blot. It provides theoretical support for targeted antiepileptic therapy and new insights into the processing of *G. elata* and the development of health foods.

## 2. Materials and Methods

### 2.1. Materials

*G. elata* medicinal materials (batch No. 231027) were purchased from Baishan City, China, and authenticated by Professor Shumin Wang. The reference medicinal material of *G. elata* (YT000901) was obtained from DST Biotech Co., Ltd., Nanjing City, China. GAS (M11GB148059) and PE (M20HB178561) were supplied by DST Biotech Co., Ltd., Nanjing City, China. *p*-HBA (R09D8X50333), PA (M09HB177711), and PB (F181B207357) were purchased from Shanghai Yuanye Biotechnology Co., Ltd., Shanghai City, China. PC (DST220810-065) was provided by the National Institutes for Food and Drug Control, Beijing City, China. High-purity methanol and acetonitrile (HPLC grade) were obtained from Fisher Scientific (Madison, WI, USA). Detailed experimental materials are provided in Supplementary Material S1.1.

### 2.2. Methods

#### 2.2.1. Animal Grouping and Drug Administration

Fifty-six SPF-grade male Sprague-Dawley (SD) rats weighing 180–220 g were provided by Liaoning Changsheng Biotechnology Co., Ltd. (animal production license: SCXK (Liao) 2024-0001) (Benxi, China). All animal procedures were approved by the Animal Ethics Committee of Changchun University of Chinese Medicine (approval No. 2024305, Approval Date: 7 June 2024). The rats were housed in a clean animal room at 18–22 °C.

After one week of adaptive feeding, 56 rats were randomly divided into 7 groups, with 8 rats in each group: Control group, Model group, Positive group (VPA), GEL (low-dose unprocessed *G. elata*), GEH (high-dose unprocessed *G. elata*), and YPGEH (high-dose YPGE). Detailed animal grouping and treatment protocols are listed in Supplementary Material S1.2.1.

#### 2.2.2. Determination of the Contents of Seven Bioactive Compounds, Including GAS, HBA, Parishins, and *G. elata* Polysaccharides (GEPs), by HPLC

The detailed procedures for the preparation of test sample solutions, reference substance solutions, and standard curves, as well as the specific determination methods, chromatographic conditions, appearance and character evaluation, and the determination method for the comprehensive score are all provided in the Supplementary Materials S1.2.2–S1.2.4 and Tables S1 and S2 [35].

#### 2.2.3. Optimization of Processing Technology for *G. elata*

The analytic hierarchy process (AHP) and entropy weight method (EWM) were applied to determine comprehensive weights for quality evaluation. Single-factor experiments were performed to investigate the effects of key parameters, followed by Box–Behnken design–response surface methodology to optimize the preparation process. Finally, the optimal process was validated, and the contents of active components between the optimized product and *G. elata* were compared. The detailed experimental protocol is provided in Supplementary Material S1.2.5.

#### 2.2.4. In Vitro Antioxidant Activity

The antioxidant activity of the optimized *G. elata* extract was evaluated by DPPH, ABTS, and hydroxyl radical scavenging assays. Briefly, different concentrations of the extract were mixed with the corresponding radical solutions, incubated at room temperature in the dark, and the absorbance was measured by a microplate reader. The IC50 value (concentration required to scavenge 50% of radicals) was calculated to evaluate the antioxidant capacity. Detailed experimental procedures and raw data are provided in Supplementary Material S1.2.6.

#### 2.2.5. Behavioral Observation

After model establishment, rats received 2-day water maze adaptation training to minimize stress-related interference. Positioning navigation and spatial search tests were performed. The detailed experimental procedures are provided in Supplementary Materials S1.2.7.

#### 2.2.6. HE Staining Observation of Hippocampal Tissue

Brain tissue was fixed in 4% paraformaldehyde. After dehydration and further processing, the tissue was embedded in paraffin, and sections were prepared at a thickness of approximately 4 μm. The sections were dewaxed, stained with hematoxylin and eosin (H&E), and mounted. Pathological alterations in the brain tissue were observed under a microscope.

#### 2.2.7. Detection of Inflammatory Cytokines, Oxidative Stress Indicators, and Neurotransmitters

An enzyme-linked immunosorbent assay (ELISA) kit (Jiangsu Enzyme Immunoassay Co., Ltd., Yancheng, Jiangsu City, China) was used. Brain tissue was harvested and weighed, and 9 volumes of PBS buffer (pH 7.3, Shanghai Biyuntian Biotechnology Co., Ltd., Shanghai City, China) were added. The mixture was homogenized completely using a homogenizer and centrifuged at 3000 r/min for 20 min. The supernatant was carefully pipetted into 0.5 mL centrifuge tubes at 100 μL per aliquot. One aliquot was prepared for immediate detection, and the remainder was frozen for subsequent analysis. The levels of TNF-α, IL-1β, GSH-P, GLU, MDA, GABA, SOD, and P-gp were detected. Detailed detection procedures are described in Supplementary Material S1.2.8.

#### 2.2.8. The Effect of YPGE on the Hippocampus of Rat Brain Tissue

Paraffin sections were taken. After routine dewaxing and rehydration, they were immersed in citric acid for 8 min, then washed with PBS buffer three times, for 5 min each time. The detailed detection procedures are shown in Supplementary Material S1.2.9.

#### 2.2.9. Western Blot Analysis

Brain tissues were homogenized in pre-cooled lysis buffer, and total protein was extracted and quantified using the BCA assay. Detailed detection procedures are shown in Supplementary Material S1.2.10.

#### 2.2.10. Statistical Analysis

The statistical values are expressed as the mean ± standard deviation (SD). Statistical comparisons were analyzed using a one-way analysis of variance (ANOVA) followed by Dunn’s test via IBM SPSS 22.0 software and GraphPad Prism 8.0 (GraphPad Software Inc., San Diego, CA, USA). *p* < 0.05 was considered to indicate a statistically significant difference.

## 3. Results

### 3.1. Standard Curves of Seven Bioactive Compounds, Including GAS, HBA, Parishins, and G. elata Polysaccharides (GEPs)

The standard curves for determining the content of each component in *G. elata* are presented in Table 1. It can be observed that GEP, GAS, *p*-HBA, and TP (PA, PB, PC, and PE) exhibit good linearity within their respective concentration ranges, making them suitable for subsequent content determination.

### 3.2. Single Factor Experimental

In this experiment, the most representative processing conditions were first selected for single-factor investigation. To achieve the organic integration of subjective experience and objective data, this study adopted a combined weighting model of AHP and EWM: AHP was used to integrate experts’ subjective cognition of the pharmacological importance of core indicators such as GEP and GAS, while the EWM was employed to quantify the objective weights of indicators based on data dispersion. The synergistic optimization of these two methods rendered the multi-index comprehensive evaluation system more scientifically rigorous. Consistency test results showed that the CR was 0.010 and 0.015, respectively (both < 0.1), confirming the logical rationality of the weight assignment and effectively avoiding the empirical dependence of single subjective weighting and the information bias of pure objective weighting. Experimental results indicated that the optimal processing parameters for YPGE were as follows: alcohol by volume of 18%, drying temperature of 75 °C, and drying time of 30 h. This optimized process provides theoretical support and technical reference for the industrial production and quality standardization of YPGE, with relevant results shown in Figure 2.

### 3.3. Box–Behnken Design–Response Surface Method

Based on the results of the single-factor experiments, a total of 17 batches of YPGE samples were prepared, and the comprehensive scores of each group of samples were obtained. The results are shown in Table 2.

The experimental data were analyzed using multiple regression and analysis of variance via Design-Expert 8.0 software, yielding the following polynomial regression equation: OD = 90.8 + 4.125A − 3.75B + 4.375C − 1.25AB + 3AC + 1.75BC − 9.15A2 − 10.4B2 − 15.15C2. Analysis of variance results (Table 3) revealed that the regression model was highly significant (*p* < 0.01), confirming its statistical validity. The lack-of-fit test, with a *p*-value of 0.0676 (non-significant), indicated minimal interference from unaccounted variables. With an R2 value of 0.9739, the model demonstrated excellent goodness of fit, justifying its reliability for predicting experimental outcomes. Within the equation, linear terms (A, B, C) and quadratic terms (A2, B2, C2) significantly influenced the response (*p* < 0.05), whereas interaction terms (AB, AC, BC) were non-significant, suggesting limited synergistic effects among factors. Based on the F-values of linear terms, the order of factor influence was: drying time > alcohol by volume > drying temperature.

The response surface and contour plots are presented in Figure 3A–F. Optimization with respect to the maximum comprehensive score indicated that the optimal processing parameters for YPGE were 18% alcohol by volume, a drying temperature of 72 °C, and a drying time of 32 h.

### 3.4. Verification of Optimal Process

Three parallel experiments were conducted using the optimized processing method. The content of each index was determined, and the comprehensive scores were calculated, with results presented in Table 4. The average comprehensive score was 90.66, with a relative standard deviation (RSD) of 1.35%. The RSD between this average and the predicted value (95) was less than 3%, indicating that the response surface optimization model has good predictability and the processing technology is stable and reliable.

### 3.5. Contents of Active Components Before and After Processing Determined by HPLC

The pharmacodynamic material basis of *G. elata* mainly relies on phenolic components and polysaccharides, which exert a synergistic effect to jointly underpin its core pharmacological activities: sedation, tranquilization, and antioxidant capacity [36,37]. The determination results of relevant component contents in GE and YPGE are presented in Figure 4A, where all data represent the mean values calculated from three parallel experiments. Compared with traditional processing methods such as steaming, sulfur fumigation, and yellow rice wine steaming, the unique feature of this study lies in the significant improvement in the contents of various bioactive components in *G. elata* after soaking in yellow rice wine [20,30]. A plausible mechanism underlying this phenomenon is that during the soaking process, *G. elata* not only absorbs certain bioactive components from yellow rice wine but also undergoes enhanced conversion of its intrinsic active ingredients under the action of the wine. Parishin exhibits potent antioxidant activity and neuroprotective effects [38]. After wine processing, its content in YPGE surges by 3.58-fold compared with GE. This phenomenon is presumably attributed to the favorable environment created by yellow rice wine soaking for parishin biosynthesis [39]. Parishin is thermolabile and most stable under pH conditions of 3–6; the acidic environment of yellow rice wine can effectively prevent its degradation. Meanwhile, trace citric acid present in yellow rice wine may react with GAS, promoting the conversion of GAS to parishin and thereby significantly increasing the content of this component [40,41,42]. As a signature phenolic bioactive component of *G. elata*, GAS is the core substance mediating its neuroprotective, sedative, and hypnotic effects. After wine processing, its content increases by 0.54-fold compared with that in GE but is significantly lower than the GAS content in *G. elata* processed by yellow rice wine stir-frying. This phenomenon may be attributed to the excessive temperature during the stir-frying process, which inhibits the activity of β-D-glucosidase and thereby reduces the conversion of GAS to *p*-HBA [42]. GEPs possess significant antioxidant, immunomodulatory, and anti-fatigue activities. Following wine processing, their content in YPGE increases by 0.13-fold compared with GE. The elevated polysaccharide content may be associated with improved solubility after wine processing. Although the increment is relatively modest, it can still enhance the efficacy of YPGE in resisting oxidative stress and improving the body’s immune status [35,43]. *p*-HBA exerts anti-inflammatory effects, protects the vascular endothelium, and improves microcirculation. After wine processing, its content in YPGE substantially increases, by 1.87-fold, compared with GE, and this change is closely related to the β-D-glucosidase-catalyzed conversion of gas to *p*-HBA [44].

### 3.6. In Vitro Antioxidant Activity Results

Figure 5A presents the results of the DPPH radical scavenging assay. As the concentration of YPGE increased from 2 mg/mL to 6 mg/mL, its DPPH radical scavenging capacity improved significantly. However, the improvement tended to plateau with further increases in concentration. At 8 mg/mL, YPGE exhibited a DPPH radical scavenging rate of 84.29%, slightly higher than that of the positive control (VC) at the same concentration (84.17%), with no significant difference observed (*p* > 0.05). This indicates that YPGE possesses a DPPH radical scavenging capacity comparable to that of VC. Additionally, the DPPH radical scavenging capacity of YPGE was significantly superior to that of GE.

Figure 5B presents the results of the ABTS radical scavenging assay. Analysis indicates that VC exhibits the most potent ABTS radical scavenging activity. Within the concentration range of 2–10 mg/mL, the scavenging rate of GE increased from 30.71% to 84.66%, while that of YPGE rose from 46.34% to 95.38%, showing a concentration-dependent enhancement (R2 > 0.90). Specifically, at concentrations of 6–10 mg/mL, YPGE displayed significantly higher scavenging capacity than GE (*p* < 0.05). At 10 mg/mL, the scavenging rate of YPGE (95.38%) differed by only 2.03% from that of the positive control VC (97.41%) at the same concentration, indicating a potent scavenging capacity comparable to VC. Although YPGE’s ABTS radical scavenging capacity was lower than VC within this range, the concentration-scavenging rate trend suggests that its activity may further increase beyond 10 mg/mL.

Figure 5C presents the results of the hydroxyl radical scavenging assay. Analysis reveals that within the concentration range of 2–10 mg/mL, the scavenging rate of GE increased from 11.59% to 63.54%, while that of YPGE rose from 21.52% to 78.90%. Across this concentration range, YPGE exhibited a significantly higher scavenging rate than GE (*p* < 0.05). Furthermore, at 10 mg/mL, YPGE showed a scavenging capacity comparable to the positive control VC, demonstrating strong hydroxyl radical scavenging activity.

Table 5 summarizes the IC50 values from the three free radical scavenging assays. Compared with GE, the IC50 values of YPGE for scavenging DPPH, ABTS, and hydroxyl radicals decreased by 1.115, 0.598, and 2.348 mg/mL, respectively. This indicates that yellow rice wine processing significantly enhances the free radical scavenging capacity of *G. elata*, with its antioxidant activity being markedly higher than that of the GE.

### 3.7. Behavioral Observation Results

The behavioral observation results (mean values within 28 days) are presented in Table 6. Differences between the drug administration group and the model group were analyzed for statistical significance. It was found that the number of convulsions significantly decreased, the seizure latency was significantly prolonged, and the seizure duration was significantly shortened. YPGEH showed the best convulsion inhibition rate, indicating that YPGE has a strong anti-epileptic effect.

### 3.8. Positioning Navigation Results

The results of the place navigation test are shown in Table 7. Groups were set as the abscissa, and escape latency(s) as the ordinate for plotting, as displayed in Figure 6. The escape latency was an important indicator for measuring the spatial learning ability of animals. Shorter escape latency suggested that animals possessed good learning and memory abilities, namely animals could learn to find the location of the hidden platform more quickly. The results showed that the model group had severe cognitive impairment (#### *p* < 0.0001); compared with the model group, both *G. elata* and YPGEs showed significantly improved cognitive function (**** *p* < 0.0001).

### 3.9. Space Search Results

The results of the spatial probe test are presented in Table 8 and Figure 7. In Figure 7, groups are set as the abscissa, and the residence time (s) in the target quadrant is taken as the ordinate for plotting. Subsequently, the differences between each drug-treated group and the model group were compared. The results showed that compared with the control group, the residence time of the model group was significantly shorter than that of the control group, indicating obvious memory impairment; compared with the model group, both *G. elata* and YPGEs showed significantly improved spatial memory (*p*-0.0003, **** *p* < 0.0001).

### 3.10. HE Staining Observation of Hippocampal Tissue Results

The results of the histopathological examination are shown in Figure 8. In the control group, in the brain tissue, the number of cortical neurons was abundant, with uniform staining, neat arrangement, and clear layering; the nucleus was large and round, appeared uniform light blue, and the nucleolus was clear; the hippocampal structure was normal; no other obvious abnormalities were found. In the brain tissue of the Model group, a small amount of neuronal shrinkage was observed in the cerebral cortex and hippocampus (black arrows), with staining intensification, and cell shrinkage and deformation; the boundary between the cell nucleus and cytoplasm was unclear; there were occasional hemorrhages around the hippocampus (yellow arrows); no other obvious abnormalities were observed. Compared with the model group, in each drug-administered group, the number of cortical neurons in the brain tissue was abundant; the staining was relatively uniform and arranged neatly, with relatively clear boundaries; the cell nucleus was large and round, and it showed uniform pale blue staining with clear nucleoli. The shrinkage of hippocampal neurons in the brain tissue was improved, and the hemorrhages around the hippocampus were effectively improved and repaired.

### 3.11. Effects of YPGE on Brain Tissue in Rats

#### 3.11.1. The Effect of YPGE on Inflammation in Rat Brain Tissue

The contents of inflammatory factors TNF-α and IL-1β in brain tissue were detected by an ELISA kit in Figure 9, and the results showed that all drug-administered groups had significantly reduced levels of inflammatory factors TNF-α and IL-1β in brain tissue (*** *p* < 0.001), blocking the neuroinflammatory pathway [45] and further inhibiting the occurrence of epilepsy. GEL showed the best inhibitory effect on TNF-α, and YPGEH showed the best inhibitory effect on IL-1β.

#### 3.11.2. The Effect of YPGE on the Antioxidant Capacity of Rat Brain Tissue

The results showed that compared with the model group in Figure 10, both *G. elata* and YPGE markedly decreased MDA content (*** *p* < 0.0001). Meanwhile, GEL, YPGEL and YPGEH all obviously elevated GSH-Px levels (** *p* < 0.01, *** *p* < 0.001). These findings suggested that they could alleviate lipid peroxidation, improve antioxidant capacity and relieve oxidative stress induced by epileptic seizures.

#### 3.11.3. The Effect of YPGE on Neurotransmitters in Rats

The results of the study showed that, compared with the model group in Figure 11, YPGEH could significantly increase the content of GABA (** *p* < 0.01), which helped enhance the inhibitory effect on neuronal excitation, thereby exerting an anti-epileptic effect. Both *G. elata* and YPGE regulated the levels of Glu and GABA neurotransmitters simultaneously and balanced the excitatory and inhibitory neurotransmitters, thus preventing and inhibiting epileptic seizures to a certain extent.

#### 3.11.4. The Effect of YPGE on Blood–Brain Barrier Factors in Rats

The expression level of P-glycoprotein (P-gp) in brain tissues was determined by ELISA in Figure 12. Compared with the control group, the level of P-gp was slightly increased in the model group, which was consistent with previous studies reporting that recurrent convulsive episodes could mildly upregulate the expression of P-gp in the blood–brain barrier [46]. Relevant studies have indicated that the upregulation of P-gp expression is not necessarily positively correlated with the antiepileptic effect, and its physiological function depends on substrate types and regulatory context.

Traditional antiepileptic drugs, such as phenobarbital, phenytoin sodium and carbamazepine, are all substrates of P-gp. Long-term administration can induce the overexpression of P-gp, thereby accelerating the efflux of drugs at the blood–brain barrier, reducing drug concentration in brain tissue and ultimately leading to drug resistance [46,47]. The results of the present experiment showed that the expression of P-gp in the positive drug valproate sodium (VPA) was slightly decreased compared with the model group. In contrast, both GE and YPGE could upregulate the expression of P-gp, and the upregulation effect of YPGE was significantly higher than that in the model group (*p* < 0.01).

This may be attributed to the fact that the active ingredients of *G. elata* are not typical substrates of P-gp. Accordingly, the YPGE-induced upregulation of P-gp does not impair its inherent antiepileptic efficacy. In contrast, as a protective efflux transporter located on the blood–brain barrier, P-gp is primarily responsible for pumping harmful substances such as endogenous neurotoxins, oxidative stress metabolites and pro-inflammatory mediators out of brain tissues. By moderately upregulating P-gp expression, YPGE enhances the defensive function of the blood–brain barrier, accelerates the clearance of harmful substances in epileptic brain tissue, alleviates secondary neuronal damage, and further exerts neuroprotective effects in synergy with its anti-inflammatory, antioxidant and anti-apoptotic activities.

In summary, the upregulation of P-gp induced by YPGE represents a protective regulation rather than an abnormal overexpression associated with drug resistance. This mechanism contributes to the improvement of the brain tissue microenvironment and participates in the overall antiepileptic effect.

### 3.12. The Effect of YPGE on the Hippocampus of Rat Brain Tissue

The TUNEL staining of the hippocampal CA1 region in rats of each group observed under a fluorescence microscope is shown in Figure 13. The cells were counted and the neuronal apoptosis rate was calculated. The neuronal apoptosis rates in the hippocampal CA1 region of the control group, model group, positive group (VPA), GEL, GEH, YPGEL and YPGEH were 29%, 81%, 59%, 42%, 34%, 33% and 31%, respectively. The apoptosis rate of the model group was significantly higher than that of the control group, suggesting that the PTZ chronic kindling model successfully induced hippocampal neuronal injury. The Positive group exhibited partial neuroprotective effects, while YPGEL and YPGEH exerted the most potent effects, which indicated that YPGEs targeted neurons and produced anti-apoptotic activity. Nevertheless, the apoptosis rate only reflected the final outcome of neuronal apoptosis and could not elucidate the specific anti-apoptotic mechanisms.

Compared with the control group, a significantly reduced number of NeuN-positive neurons and severe neuronal loss were observed in the model group. Positive group treatment effectively ameliorated neuronal loss. The number of NeuN-positive neurons in the GEL group was significantly increased compared with that in the model group. Compared with the GEH group, the YPGEL and YPGEH groups exhibited a greater number of NeuN-positive neurons and more intact neuronal morphology. Notably, the YPGEH group exerted the most prominent protective effect.

### 3.13. The Mechanism of YPGE in Antiepileptic Action

Western Blot was used to explore the mechanism underlying the regulatory effect of YPGE on neuronal apoptosis in the hippocampal CA1 region. Image J 1.54g software was applied to detect the gray values of internal reference and target protein bands [48], and the relative protein expression levels were calculated. The relevant results are presented in Figure 14.

Bcl-2 acts as an apoptosis inhibitor and suppresses apoptosis in a variety of cell systems. The mitochondrial pathway serves as the major pathway of cellular apoptosis [49], and Bcl-2 family proteins are the core regulatory molecules of the mitochondrial apoptotic pathway [50]. Their expression levels directly determine the scope of cell apoptosis. Detection of the expression level of Bcl-2 can clarify whether *G. elata* exerts its neuroprotective effect on the hippocampal CA1 region by up-regulating the anti-apoptotic protein Bcl-2 or down-regulating the pro-apoptotic protein (Bax) [51].

In Figure 15, compared with the control group, the protein level of Bcl-2 in the model group was significantly decreased (#### *p* < 0.0001). Both GE and YPGE markedly upregulated Bcl-2 expression (**** *p* < 0.0001), among which YPGEH showed the most prominent effect. As a member of the Bcl-2 family, Bax acts as a key regulator of cell apoptosis. Compared with the control group, the protein expression of Bax was obviously increased in the model group (#### *p* < 0.001). In contrast, both GE and YPGE significantly downregulated Bax expression (*** *p* < 0.001, **** *p* < 0.0001), and YPGEH exerted a relatively stronger regulatory effect. These findings indicated that rice wine-processed *G. elata* could modulate the expression of Bcl-2 family proteins, downregulate Bax expression and stabilize mitochondrial membrane potential, thereby inhibiting neuronal apoptosis in the hippocampal CA1 region and alleviating neuronal damage induced by epilepsy [52].

## 4. Discussion

Aiming at the deficiencies of insufficient research depth, lack of scientific quantification and single evaluation system in the traditional rice wine processing technology of *G. elata*, this study for the first time integrated the analytic hierarchy process and entropy weight method for combined subjective and objective weighting, in conjunction with the Box–Behnken design–response surface methodology, to establish a multi-index and unified subjective-objective optimization system for YPGE. The optimal processing conditions were determined as follows: an alcohol content of 18%, a drying temperature of 72 °C, and a drying duration of 32 h. This system effectively compensates for the deficiencies of traditional processing, which relies heavily on empirical experience and is difficult to standardize. This method exhibits superior scientific rationality, stability and reproducibility and achieves precise matching between processing parameters and comprehensive quality evaluation. It provides a quantifiable, modern and standardized methodological model for the processing of *G. elata*.

Through systematic comparison with raw *G. elata*, this study firstly confirmed that rice wine processing significantly elevated the contents of GAS, GRP, *p*-HBA and TP and improved the in vitro antioxidant capacity of *G. elata*. The IC values of DPPH, ABTS and hydroxyl radical scavenging activities were 2.604 mg/mL, 2.719 mg/mL and 4.046 mg/mL, respectively. Such increases in the levels of these active ingredients provided a direct material basis for the enhanced antioxidant and antiepileptic effects of YPGE.

Systematic evaluation of in vivo antiepileptic effects was performed using a PTZ-kindled epileptic rat model. Compared with GE, YPGE exerted superior antiepileptic and neuroprotective activities. Using the Morris water maze as a core evaluation tool for cognitive function [53,54], the memory and cognitive functions of rats were evaluated through two indicators: “escape latency” and “target quadrant residence time”. The results showed that this experiment can not only intuitively reflect the neuroprotective effect but also provide relevant evidence for subsequent mechanism studies. Behavioral tests and Morris water maze assays revealed that both GE and YPGE could ameliorate epileptic behaviors in rats to varying degrees, as evidenced by reduced seizure frequency and prolonged seizure latency. Moreover, both treatments effectively improved spatial cognitive ability, characterized by shortened escape latency and increased target quadrant residence time. Histopathological and biochemical indicator analyses further demonstrated that both GE and YPGE could alleviate hippocampal damage and maintain the structural integrity of hippocampal neurons to varying degrees. Meanwhile, they downregulated the levels of pro-inflammatory cytokines (TNF-α, IL-1β) in brain tissue, ameliorated oxidative stress by increasing SOD and GSH-Px activities while decreasing MDA content, and regulated the balance of neurotransmitters through reducing Glu level and elevating GABA level. YPGE also significantly upregulated the expression of P-glycoprotein (*p* < 0.01). Its antiepileptic and neuroprotective effects may be achieved by enhancing the defensive function of the blood–brain barrier and eliminating toxic substances in the brain. This upregulation differs from the drug resistance-related upregulation induced by antiepileptic drugs. It does not result in enhanced efflux that weakens therapeutic efficacy but represents a neuroprotective regulatory pattern.

YPGE inhibited the mitochondrial apoptotic pathway in the hippocampal CA1 region of rats [55] (the neuronal apoptosis rate was significantly decreased), synergistically scavenged free radicals (the levels of SOD and GSH-Px were significantly increased, and the level of MDA was significantly decreased), regulated neuroinflammation [56] (TNF-α and IL-1β were significantly decreased), maintained neurotransmitter balance (the level of glutamate was significantly decreased, and GABA showed an upward trend), significantly improved behavior during epileptic seizures (the number of convulsions was reduced, the latency was prolonged) and improved spatial cognitive function (the escape latency was shorter, more time was spent in the target quadrant).

Therefore, it was inferred that the activities of SOD and GSH-Px were increased and the content of malondialdehyde (MDA) was decreased in rats of the administration group, which alleviated lipid peroxidation [51], maintained the integrity of the mitochondrial membrane and inhibited the activation of Bax, thereby lowering the cell apoptosis index. Meanwhile, the downregulation of TNF-α and IL-1β suppressed the NF-κB pathway, which further reduced the expression of pro-apoptotic genes and elevated the Bcl-2/Bax ratio. Collectively, these effects jointly constitute an anti-epileptic mechanism mediated by the synergistic action of anti-inflammatory, antioxidant and anti-apoptotic activities.

Collectively, this study successfully optimized the processing protocol of rice wine-processed *G. elata* via AHP-EWM combined with BBD-RSM, which markedly improved the bioactive component accumulation and enhanced the antiepileptic pharmacological efficacy of YPGE. Through systematic comparison with GE, this study confirmed that rice wine processing is an effective strategy to enhance the antiepileptic effect of *G. elata*.

Nevertheless, several limitations of the present study should be acknowledged. (1) The animal model adopted in this study is relatively singular. Only the PTZ-kindled epileptic model was used, which cannot fully cover the complex etiologies and pathological types of clinical epilepsy. (2) The mechanistic exploration remains relatively limited. Only conventional pathways including inflammation, oxidative stress and apoptosis were validated, while further investigations on in-depth signaling pathways, target identification and molecular interaction were not performed.

## 5. Conclusions

This study established and validated the optimal processing technology for rice wine-processed *G. elata* based on the combined weighting of AHP-EWM coupled with Box–Behnken response surface methodology. The established process is stable and reliable and can significantly elevate the contents of bioactive components and enhance the antioxidant capacity of *G. elata*.

Compared with GE, YPGE exhibited superior effects in antiepilepsy, neuroprotection, cognitive improvement, anti-inflammation, antioxidation and anti-apoptosis in the PTZ-kindled epileptic rat model. It is confirmed that rice wine processing can markedly strengthen the antiepileptic efficacy of *G. elata*.

This study provides a scientific basis and technical support for the standardized processing, quality control, and development of medicinal and edible products of *G. elata*.

## Figures and Tables

**Figure 1:**
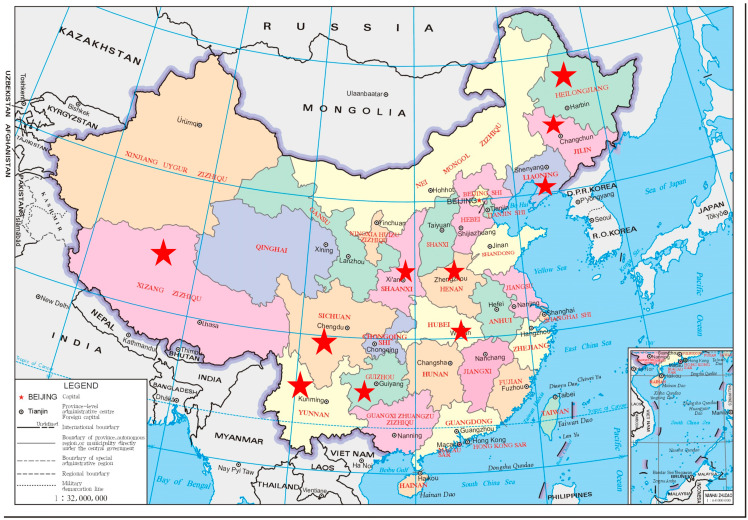
Geographical distribution map of *G. elata.* Red stars mark the primary geographic distribution of *G. elata* in China. Source: Standard Chinese administrative boundary map obtained from official public domain database.

**Figure 2:**
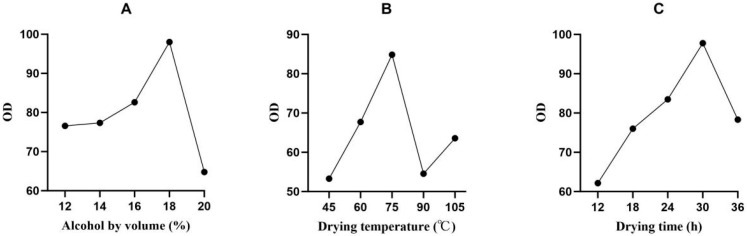
Effects of different processing conditions on the scoring index of YPGE. (**A**) Effect of alcohol by volume on the score value. (**B**) Effect of drying time on the score value. (**C**) Effect of drying temperature on the score value.

**Figure 3:**
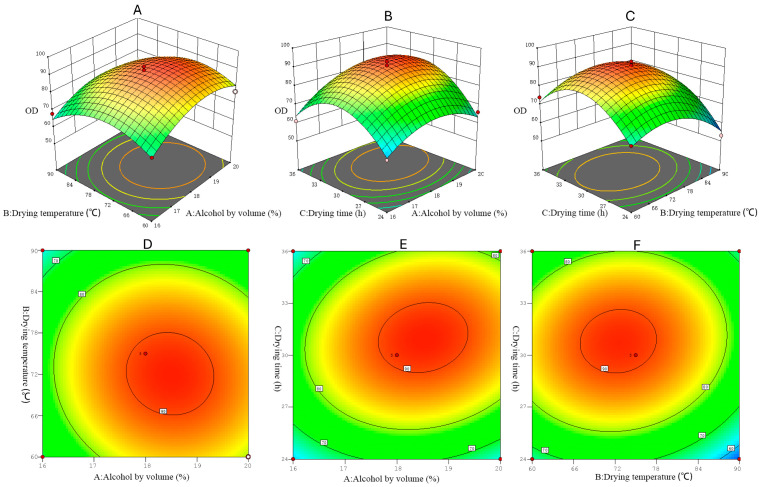
3D response surfaces and contour plots of interaction between various factor results. (**A**) 3D response surface plot of interactive alcohol volume fraction A and drying temperature B on OD. (**B**) 3D response surface plot of interactive alcohol volume fraction A and drying time C on OD. (**C**) 3D response surface plot of interactive drying temperature B and drying time C on OD. (**D**) Contour plot for alcohol volume fraction A and drying temperature B interaction on OD. (**E**) Contour plot for alcohol volume fraction A and drying time C interaction on OD. (**F**) Contour plot for drying temperature B and drying time C interaction on OD.

**Figure 4:**
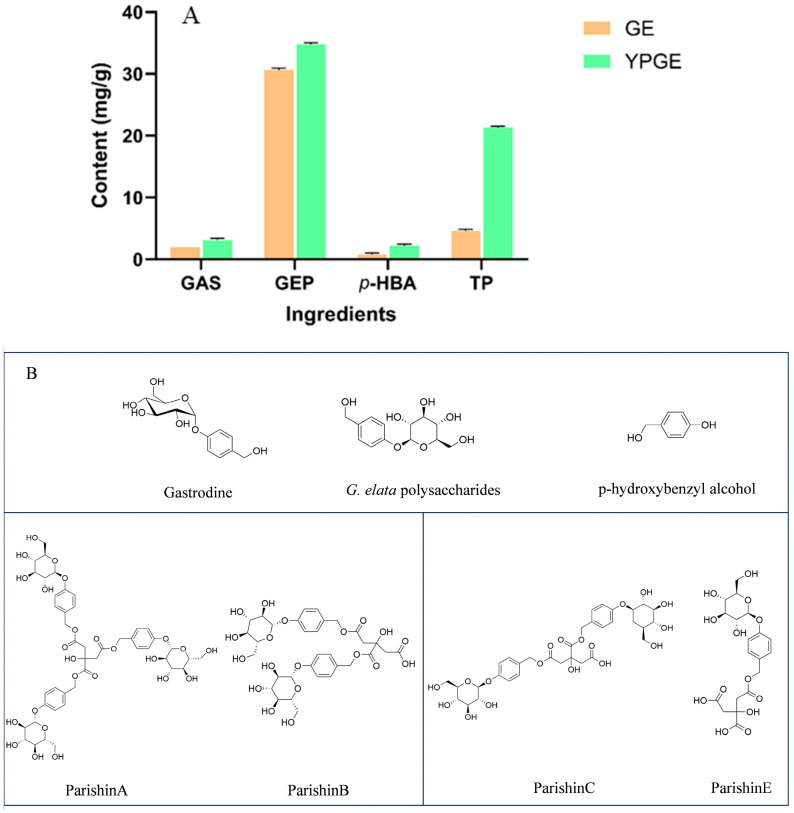
(**A**) The determination of the main active ingredient contents of *G. elata* and YPGE, versus *G. elata*. (**B**) Small-molecule metabolite chemical structure.

**Figure 5:**
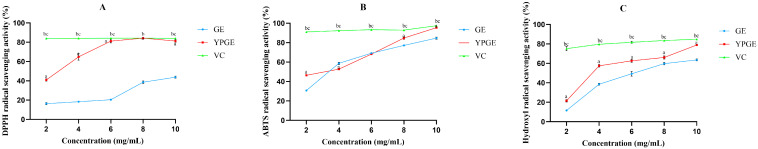
Antioxidant activities of GE and YPGE: (**A**) DPPH radical scavenging activities of GE and YPGE. (**B**) ABTS radical scavenging activities of GE and YPGE. (**C**) Hydroxyl radical scavenging activities of GE and YPGE. “a” indicates a significant difference between GE and YPGE at the same concentration. “b” indicates a significant difference between GE and VC at the same concentration. “c” indicates a significant difference between YPGE and VC at the same concentration (*p* < 0.05).

**Figure 6:**
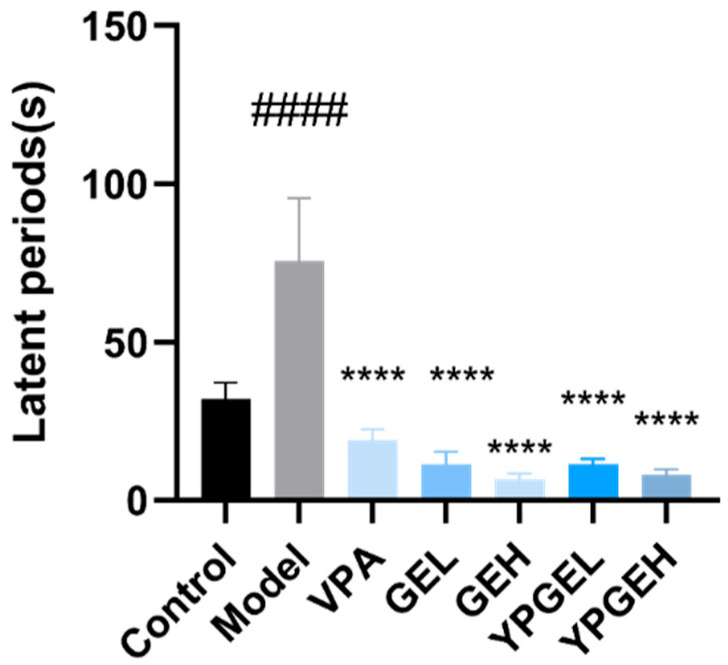
The effects of different rat groups on cognitive impairment, **** *p* < 0.0001 versus the model group, #### *p* < 0.0001 compared with the control group.

**Figure 7:**
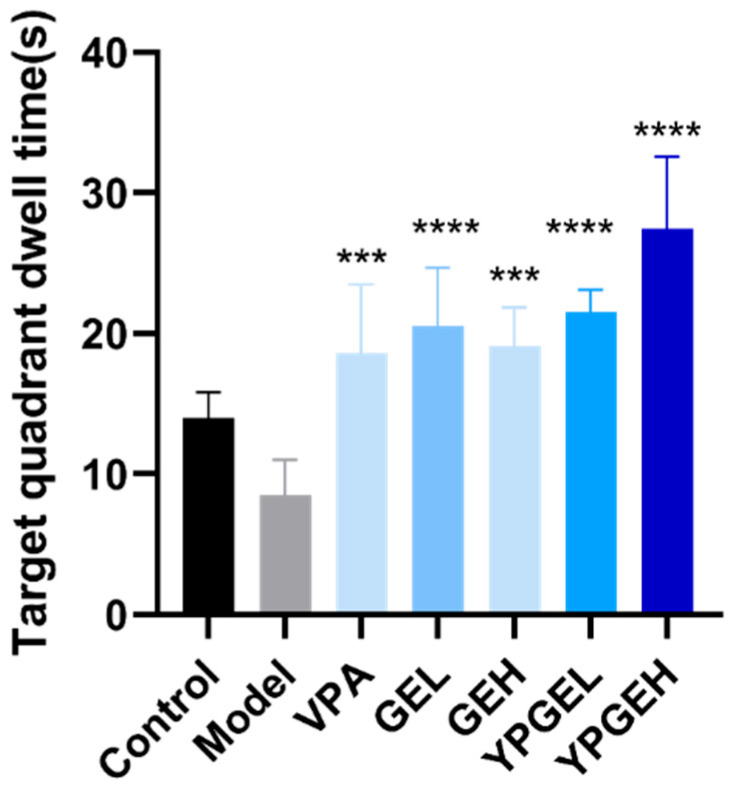
The effects of different rat groups on the residence time in target quadrant of spatial probe, *** *p* < 0.001, **** *p* < 0.0001 versus the model group.

**Figure 8:**
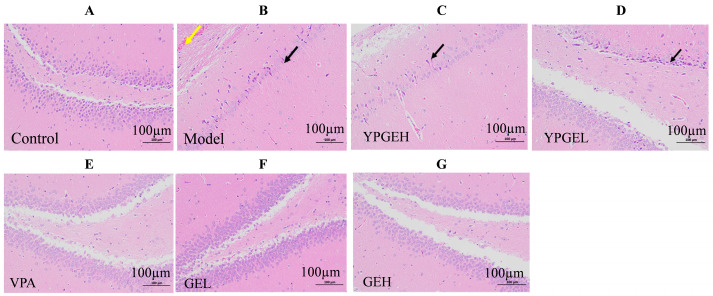
The effect of YPGE on the hippocampus of rat brain tissue. Histopathological changes are shown following H&E staining (magnification, ×200). (**A**) Control group: intact neuronal structure. (**B**) Model group: neuronal shrinkage (black arrow) and perihippocampal hemorrhage (yellow arrow). (**C**–**G**) Drug-treated groups: (**C**) YPGEH, (**D**) YPGEL, (**E**) VPA, (**F**) GEL, (**G**) GEH. All treatments attenuated neuronal injury and hemorrhage compared to the model group.

**Figure 9:**
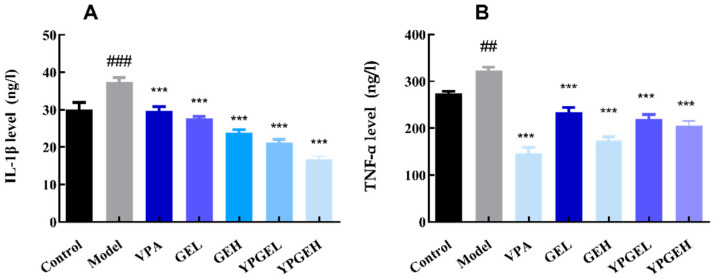
The effect of YPGE on TNF-α and IL-1β levels was measured using an ELISA kit. (**A**): TNF-α, (**B**): IL-1β. Compared with the model group, *** *p* < 0.001. Compared with the control group, ## *p* < 0.01, ### *p* < 0.001.

**Figure 10:**
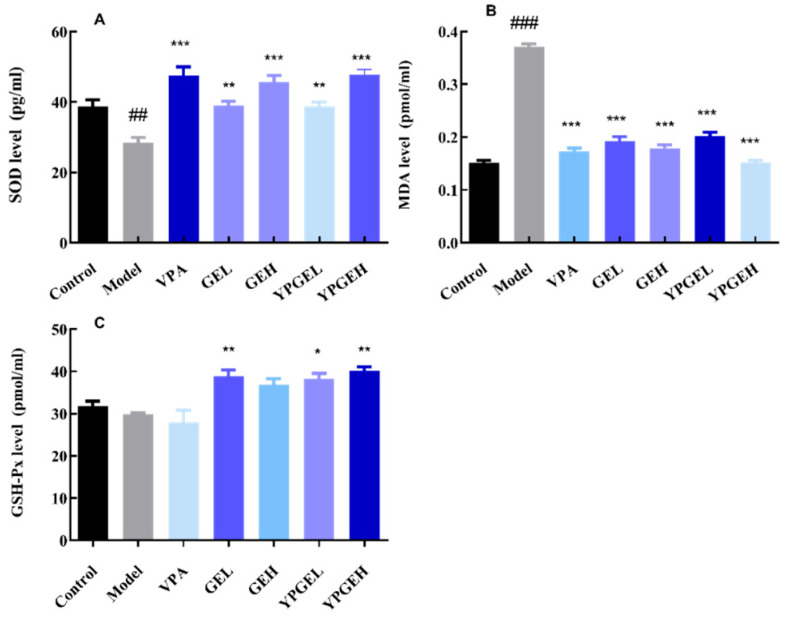
The effect of YPGE on SOD, MDA and GSH-Px levels was measured using an ELISA kit. (**A**): SOD, (**B**): MDA, (**C**): GSH-Px. Compared with the model group, * *p* < 0.05, ** *p* < 0.01, *** *p* < 0.001; compared with the control group, ## *p* < 0.01, ### *p* < 0.001.

**Figure 11:**
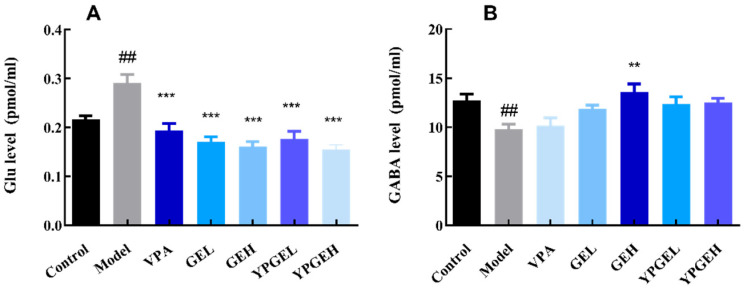
The effect of YPGE on Glu and GABA levels was measured using an ELISA kit. (**A**): Glu, (**B**): GABA. Compared with the model group, ** *p* < 0.01, *** *p* < 0.001; compared with the control group, ## *p* < 0.01.

**Figure 12:**
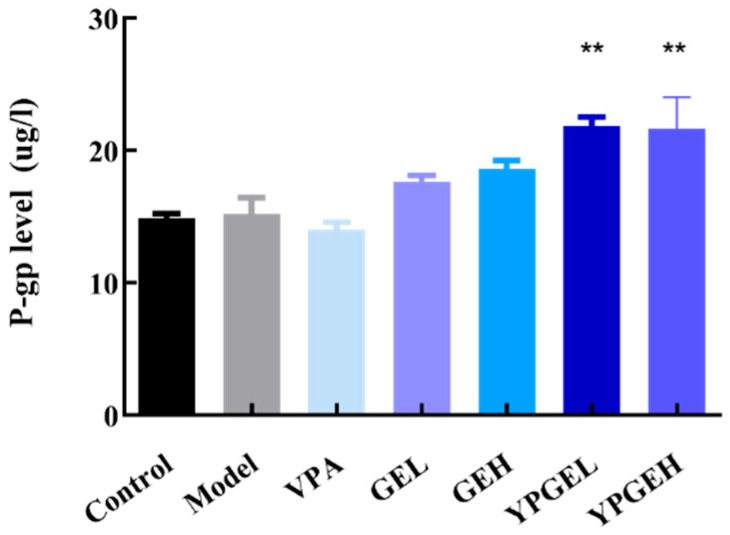
The effect of YPGE on P-gp level was measured using an ELISA kit, compared with the model group ** *p* < 0.01.

**Figure 13:**
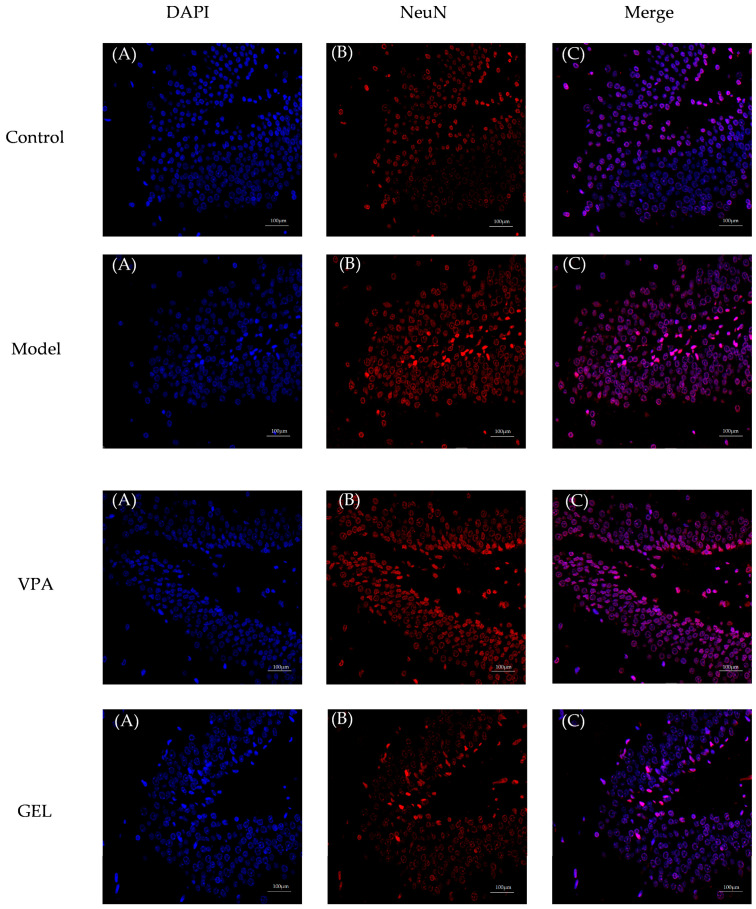

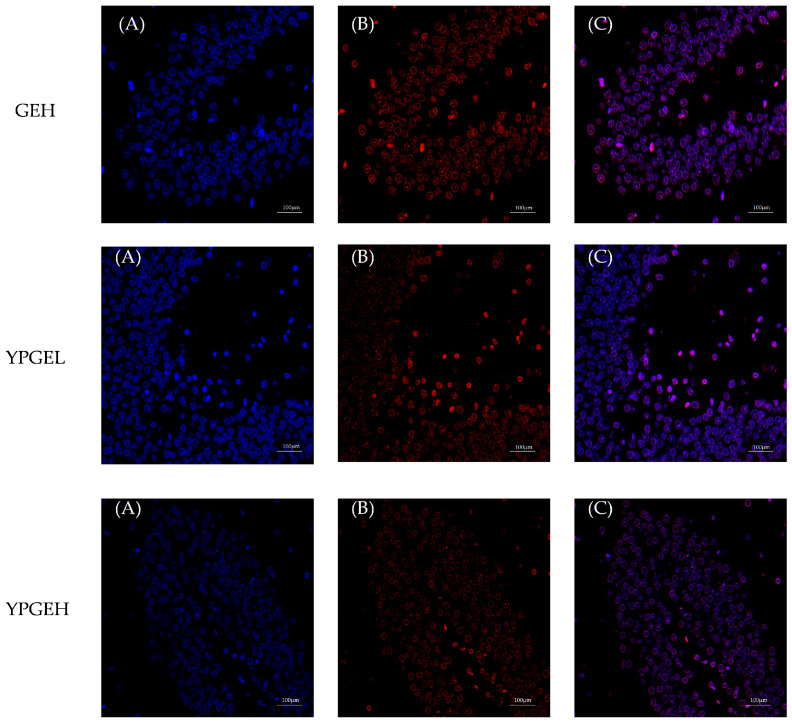
The Effect of yellow rice wine processing on neuronal apoptosis in the hippocampal CA1 region of rats. (**A**) DAPI staining (blue, cell nucleus); (**B**) NeuN staining (red, neuron-specific marker); (**C**) merged images of DAPI and NeuN staining.

**Figure 14:**
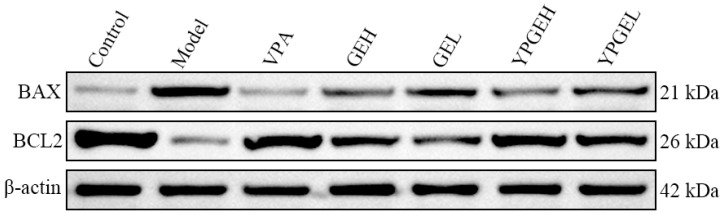
Expression levels of Bax and Bcl-2.

**Figure 15:**
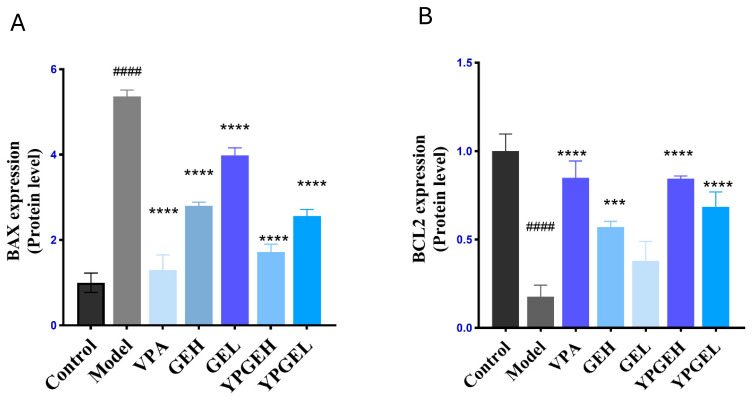
(**A**) The Effect of YPGE on the apoptotic protein Bax. Compared with the model group, #### *p* < 0.0001, compared with the control group, **** *p* < 0.0001. (**B**) The Effect of YPGE on the apoptotic protein Bcl-2. Compared with the model group, #### *p* < 0.0001; compared with the control group, **** *p* < 0.0001, *** *p* < 0.001.

**Table 1:** Standard curves of seven bioactive compounds, including GAS, HBA, parishins, and GEP.

Indicators	Regression Equation	r^2^	Linear Range(µg·mL^−1^)
GEP	y = 0.0117x − 0.0037	0.9989	10–60
GAS	y = 15,091x − 10,341	0.9996	8.34–18.56
*p*-HBA	y = 22,130x + 4638	0.9996	4.40–14.09
PE	y = 7967.4x + 131,246	0.9992	55.38–80.16
PB	y = 6103.3x + 86,173	0.9990	30.35–80.22
PC	y = 11,954x − 4954.9	0.9993	1.51–11.36
PA	y = 15,099x + 2251.1	0.9993	10.54–60.73

**Table 2:** Experimental design and results of response surface methodology for YPGE.

Number	Alcohol by Volume (%)	Drying Temperature (°C)	Drying Time (h)	OD
1	18	60	24	67.06
2	20	75	24	66.35
3	18	75	30	93.36
4	16	75	24	60.87
5	18	75	30	88.35
6	16	75	36	61.64
7	18	60	36	74.27
8	18	90	36	67.26
9	16	90	30	68.95
10	18	75	30	91.26
11	18	90	24	53.24
12	18	75	30	91.25
13	18	75	30	91.74
14	20	90	30	70.24
15	20	60	30	77.25
16	16	60	30	70.25
17	20	75	36	79.29

**Table 3:** Results of analysis of variance.

Project	Sum of Squares	df	Mean Square	F-Value	*p*-Value
model	2425.19	9	269.47	29.00	0.0014
A	136.13	1	136.13	14.65	0.0065
B	112.50	1	112.50	12.11	0.0103
C	153.13	1	153.13	16.48	0.0048
AB	6.25	1	6.25	0.6726	0.4392
AC	36.00	1	36.00	3.87	0.0897
BC	12.25	1	12.25	1.32	0.2886
A^2^	352.52	1	352.52	37.93	0.0005
B^2^	455.41	1	455.41	49.01	0.0002
C^2^	966.41	1	966.41	103.99	0.0041
Residual	65.05	7	9.29		
Lack of Fit	52.25	3	17.42	5.44	0.0676
Pure Error	12.80	4	3.20		
Cor total	2490.24	16			
model	2425.19	9	269.47	29.00	0.0014

**Table 4:** Contents of active components (mg/g) and comprehensive scores of *G. elata* in verification of optimal process.

Number	Character Score	GAS	GEP	*p*-HBA	PE	PB	PC	PA	OD
1	7	3.251	154.575	2.343	19.798	19.338	1.978	11.43	89.05
2	6	2.728	187.170	1.753	13.868	13.153	1.355	7.915	92.01
3	7	2.885	181.495	2.775	23.921	19.458	1.785	9.135	90.95

**Table 5:** IC50 values for radical scavenging capacity before and after processing.

IC50	DPPH Radical	ABTS Radical	Hydroxyl Radical
GE (mg/mL)	3.719	3.317	6.394
YPGE (mg/mL)	2.604	2.719	4.046

**Table 6:** List of behavioral observations.

	The Number of Convulsions (times/min)	Seizure Latency (min)	Seizure Time	Inhibition Rate (%)
Control	0		0	
Model	15.82 ± 5.28	2.37 ± 3.26	40.67 ± 4.89	
VPA	10.36 ± 4.67 *	6.78 ± 4.27 *	25.58 ± 4.76 **	34.51
GEL	9.48 ± 4.28 *	10.28 ± 4.57 **	20.67 ± 5.37 **	40.08
GEH	7.56 ± 4.92 **	10.79 ± 4.92 **	20.99 ± 6.02 **	52.21
YPGEL	7.26 ± 5.27 **	12.29 ± 3.57 **	15.77 ± 7.56 **	54.11

Compared with the model group, * *p* < 0.05 indicates a statistically significant difference, and ** *p* < 0.01 indicates an extremely significant difference.

**Table 7:** Positioning, navigation, evasion, incubation period(s).

Rat Numbering	Control	Model	VPA	GEL	GEH	YPGEL	YPGEH
1	39.6	72.8	15	12.5	5.2	12	10.1
2	28.9	92.7	17	9.4	8.9	10.1	7.5
3	32.3	53.3	17.5	7.8	4.2	11.6	8.4
4	33.1	65	16.6	18.7	8.5	10	6.7
5	24.1	105.9	23	9.1	6.2	14.2	6.2

**Table 8:** Space search target quadrant dwell time(s).

Rat Numbering	Control	Model	VPA	GEL	GEH	YPGEL	YPGEH
1	14.5	11	22.1	18.8	20.8	20.9	28.1
2	15.5	8	10.4	18.5	20.6	23.6	29.5
3	13.5	11	23.6	23.1	16.7	22	35.4
4	15.1	4.6	17.3	27.5	23	19.4	27.4
5	10.5	6.7	16.6	15.6	17.2	22.9	20.2

## Data Availability

The original contributions presented in this study are included in the article/Supplementary Material. Further inquiries can be directed to the corresponding authors.

## Supplementary Materials

The following supporting information can be downloaded at: https://www.mdpi.com/article/10.3390/cimb48070688/s1. References [18,33,35,43,57,58,59,60,61,62,63,64,65,66,67,68,69,70] are cited in the supplementary materials.
