# Supplementary material for: Optimized Preparation of Gastrodiae elata Extract Enhances Antiepileptic Effects by Regulating Neuroinflammation, Oxidative Stress, and Neuronal Apoptosis in Rats
Source: Curr Issues Mol Biol. 2026 Jul 3;48(7):688. doi: 10.3390/cimb48070688 (PMC13409452; doi:10.3390/cimb48070688)
Supplement: Supplementary file 1 [file cimb-48-00688-s001.zip › cimb-4276498-supplementary.pdf]

Supplementary Materials for: **Optimized Preparation of *Gastrodiae elata* Extract Enhances Antiepileptic Effects by Regulating Neuroinflammation, Oxidative Stress, and Neuronal Apoptosis in Rats**

S1. Materials and Methods

S1.1 Materials

Anhydrous ethanol was purchased from Guangdong Xilong Chemical Co., Ltd. Phosphoric acid (analytical grade) was used as received. Yellow rice wines with different alcohol contents were obtained from Shaoxing Baolongshan Wine Co., Ltd., Suzhou Sanjian Brewing Co., Ltd., Shaoxing Shengyushan Wine Co., Ltd., Zhejiang Shengta Shaoxing Wine Co., Ltd., and Zhejiang Guyuelongshan Shaoxing Wine Co., Ltd.

1,1-Diphenyl-2-picrylhydrazyl (DPPH), potassium persulfate, and pentylenetetrazol (PTZ) were purchased from Shanghai Macklin Biochemical Technology Co., Ltd. and Shanghai Aladdin Biochemical Technology Co., Ltd., respectively. 2,2′-Azino-bis (3-ethylbenzothiazoline-6-sulfonic acid) (ABTS) was obtained from Shandong Keyuan Biochemical Co., Ltd. Salicylic acid and ferrous sulfate were supplied by Tianjin Guangfu Fine Chemical Research Institute. L-Ascorbic acid was purchased from Tianjin Xinbotte Chemical Co., Ltd.

ELISA kits for TNF-α, IL-1, GSH-Px, GLU, MDA, GABA, SOD, β-actin, and P-gp were obtained from Jiangsu Enzyme Immunoassay Industry Co., Ltd. BCL-2 antibody was purchased from Proteintech (USA). PMSF and RIPA lysis buffer were supplied by Beijing Solarbio Science & Technology Co., Ltd.

Instrument: A Thermo U3000 High-Performance Liquid Chromatograph and a Shimadzu LC-20AT High-Performance Liquid Chromatograph were used in this study. Other instruments included an FW-400A High-Speed Universal Pulverizer, BT25s Electronic Analytical Balance, ZRD-A5210 Blast Drying Oven, HH-8 Digital Display Constant-Temperature Water Bath, UV-4802H UV-Visible Spectrophotometer, TGL16M Desktop High-Speed Refrigerated Centrifuge, AS Ultrasonic Cleaner, and M200 Pro Multifunctional Microplate Reader. Pathological and molecular biological instruments included a YYS-100E biological microscope, GJH-6874 pathological microtome (Leica), HCGC-757 tissue homogenizer, HHU-8364 embedding machine, and JY-300 electrophoresis apparatus. Experimental animals: A total of 56 SPF-grade male SD rats were used in this experiment, with a body weight of 180-220g. Provided by Liaoning Changsheng Biotechnology Co., Ltd., the animal production certificate number is SCK (Liao) (2024-0001). The animal experiments were approved by the Animal Ethics Committee of Changchun University of Chinese Medicine, with the approval number 2024305. The rats were housed in a clean animal room at a temperature of 18-22°C.

S1.2 Methods

S1.2.1 Animal Grouping and Drug Administration

Respectively weigh a quantitative amount of *G. elata* and YPGE, add 10 times the amount of water, decoct with strong fire for half an hour, filter and retain the medicinal liquid. Decoct the

dregs with 8 times the amount of water with slow fire for half an hour, filter, combine the two medicinal liquids, and concentrate into an extract.

According to the pharmacopoeia, the adult clinical dosage of 6 g is set as the low-dose group, and 12 g as the high-dose group. Calculated by body surface area method, for rats, 0.54g/kg is set as the low-dose group and 1.08g/kg as the high-dose group. Therefore, after converting the concentration, the extract is diluted with distilled water to prepare a medicinal solution containing 0.1 g of decocted herbal pieces per 1 ml. This results in a drug concentration of 0.01 g/ml for the low-dose group; similarly, the drug concentration for the high-dose group is converted to 0.02g/ml. Powder the sodium valproate(VPA) tablets[1], dilute with distilled water to 0.02g/ml to obtain the medicinal liquid for the Positive group, PTZ powder is diluted with normal saline to 7mg/ml to obtain the modeling solution.

After 5 days of adaptive feeding, Positive group, GEL, GEH, YPGEL (low-dose YPGE), YPGEH administer the corresponding medicinal liquid at 1 ml daily by gavage, and half an hour after gavage, Control group, GEL, GEH, YPGEL, YPGEH and Positive group all were intraperitoneally injected with 1 ml of PTZ solution; The Control group was intraperitoneally injected with 1 ml of normal saline. The modeling was completed after 28 consecutive days.

S1.2.2 Method for Determination of G. elata Polysaccharides Content

The determination of GEP content was performed with reference to the experimental methods in relevant research literature[1,2,3], and with slight modifications.

Weigh 3 g of YPGE powder, add 150 mL of petroleum ether, and heat under reflux for 1 hour. Filter and wash the residue three times with 10 mL of hot petroleum ether. Add 200 mL of 10% ethanol, heat under reflux for another hour, then filter and wash the residue three more times. Combine the filtrates, transfer to a 250 mL volumetric flask, dilute to volume, shake, and set aside.

S1.2.2.1 Preparation of reference substance solution

Precisely weigh 100 mg of anhydrous glucose and prepare a glucose solution with a concentration of 1 mg/mL. Then prepare a series of reference substance solutions with concentrations of 10, 20, 30, 40, 50, and 60 μg/mL respectively.

S1.2.2.2 Preparation of standard curve

Take 1 mL of each of the prepared reference substance solutions, add 4 mL of 0.2% anthrone-sulfuric acid solution, shake well, place in a boiling water bath for 10 min, remove and cool to room temperature. Measure the absorbance at a wavelength of 490 nm, and plot the standard curve for GEP.

S1.2.2.3 Determination method

Take 1 mL of the test sample solution, measure its absorbance according to the method described above, and calculate the GEP content based on the standard curve.

S1.2.3 Method for Determination of the Content of 6 Phenolic Components in G. elata

The content of six phenolic components in *G. elata* was determined based on the experimental methods of [4]and[3], with slight modifications.

S1.2.3.1 Chromatographic conditions

An Agilent HC-C18 column (4.6 mm×250 mm, 5 μm) was used, using 0.1% formic acid aqueous solution (A) and 0.1% formic acid in acetonitrile (B) as mobile phases, with gradient elution carried out under the conditions in Table S1 below. The column temperature was 30℃, the detection wavelength was 220 nm, the flow rate was 1.0 mL/min, the injection volume was 10 μL, and the number of theoretical plates was not less than 5000.

Table S1. Gradient elution table of mobile phase

Time (min)	A% (0.1% formic acid aqueous solution)	B% (0.1% formic acid acetonitrile)
0	85	15
7	80	20
13	70	30
23	27	31
27	62	38
31	55	45
35	50	50
40	45	55
45	42	58
50	60	40
60	90	10

S1.2.3.2 Preparation of test sample solution

Precisely weigh 2 g of YPGE medicinal material powder, accurately add 25 mL of 50% ethanol[4], and perform ultrasonic extraction for 30 min under the conditions of 300 W power and 25 Hz frequency. Cool to room temperature, filter through filter paper, measure 10 mL of the filtrate into an evaporating dish and evaporate to dryness. Dissolve the residue with 3% acetonitrile aqueous solution, transfer to a volumetric flask and dilute to 25 mL, centrifuge at 8000 rpm, and pass the supernatant through a 0.22 μm filter membrane to obtain the test sample solution.

S1.2.3.3 Preparation of reference substance solution

Precisely weigh appropriate amounts of reference substances GAS, p-HBA, PE, PB, PC, and PA, dissolve them in chromatographic-grade methanol solution, and dilute to prepare reference substance solutions with concentrations of 0.0215 mg/mL for GAS, 0.0261 mg/mL for p-HBA, 0.0225 mg/mL for PE, 0.0246 mg/mL for PB, 0.0285 mg/mL for PC, and 0.0262 mg/mL for PA, respectively.

S1.2.3.4 Preparation of standard curve

Prepare GAS standard solutions at concentrations of 8, 10, 12, 14, 16, and 18 μg/mL; p-HBA standard solutions at 4, 6, 8, 10, 12, and 14 μg/mL; PE standard solutions at 55, 60, 65, 70, 75, and 80 μg/mL; PB standard solutions at 30, 40, 50, 60, 70, and 80 μg/mL; PC standard solutions at 1, 3, 5, 7, 9, and 11 μg/mL; and PA standard solutions at 10, 20, 30, 40, 50, and 60 μg/mL, respectively. Determine the peak areas of each reference substance under the chromatographic conditions described above. Perform linear regression with the mass concentration as the abscissa (x) and the corresponding peak area as the ordinate (y) to plot the standard curves for the six phenolic components in *G. elata*.

Inject 10 μL of the test sample solution and calculate the mass concentration based on the corresponding peak area using the standard curve.

S1.2.4 Appearance and Character Evaluation

Using odor and color as evaluation indicators and aligning with the 2020 Chinese Pharmacopoeia's description of *G. elata*, historical YPGE processing experience, and input from various staff, the appearance and character evaluation criteria are detailed in Table S2.

Table S2. Evaluation criteria for the appearance and characteristics of YPGE

Characteristics	Description	Score
Color	Black	1
	Yellow	3
	Brown	5
Smell	Burnt smell	1
	Burnt aroma	3
	Burnt aroma with wine fragrance	5

S1.2.5 Determination of Comprehensive Score

S1.2.5.1Analytic Hierarchy Process

The AHP is a decision-making methodology that disaggregates complex multi-objective decision-making problems into hierarchical levels, grounded in the decision-maker's subjective insights and empirical evaluations, facilitating both qualitative and quantitative analyses[6][7]. In the context of evaluating the quality of *G. elata*, it is noteworthy that while the 2020 Edition

of the Chinese Pharmacopoeia specifies content determination solely for GAS and p-HBA under the *G. elata* category, the characteristic map analysis encompasses six components: GAS, p-HBA, PA, PB, PC, and PE[5]. Consequently, the inclusion of parishins as evaluation indicators in the quality assessment of *G. elata* is advocated to provide a more comprehensive reflection of its quality. Furthermore, appearance and characteristics are pivotal considerations for commercially available *G. elata* decoction pieces, with GEP being a critical indicator for the quality control of these decoction pieces[8]. Therefore, these 8 indicators were selected as the evaluation criteria for the single factor experiment and BBD-RSM in this study. Based on the importance of each factor and combined with AHP, the weight order of the quality evaluation indicators for *G. elata* decoction pieces was established as follows: GAS > GEP = p-HBA > PB = PA = appearance and characteristics > PC > PE. The 1-9 scaling method was used to construct a pairwise comparison priority judgment matrix, and the weights νj were calculated. The obtained weights are shown in Table 3. The consistency index (CI) of the matrix is 0.015, and the consistency ratio (CR) is 0.010, both of which are less than 0.1, indicating that the constructed matrix has consistency.

Table S3. Judgment matrix and relative scores of process indicators.

Indicators	GAS	GEP	*p*-HBA	PE	PB	PC	PA	Appearance and character	ν_j
GAS	1	2	2	7	3	5	3	3	0.284
GEP	0.5	1	1	5	2	3	2	2	0.168
p-HBA	0.5	1	1	5	2	3	2	2	0.168
PE	0.143	0.2	0.2	1	0.2	0.5	0.2	0.2	0.028
PB	0.333	0.5	0.5	5	1	2	1	1	0.100
PC	0.2	0.333	0.333	2	0.5	1	0.5	0.5	0.053
PA	0.333	0.5	0.5	5	1	2	1	1	0.010
Appearance and character	0.333	0.5	0.5	5	1	2	1	1	0.010

S1.2.5.2 Entropy Weight Method

EWM, an objective weighting method, determines weights according to the coefficient of variation of each indicator, and its evaluation process is highly repeatable[9]. In this study, deviation normalization was applied to the data. The raw data were first standardized using Formula (S1), where Yij denotes the standardized value of the i-th trial for the j-th indicator, and Xij denotes the measured value of the j-th indicator in the i-th experiment.

$$Y_{ij} = \left(X_{ij} - minX_{ij}\right)/\left(maxX_{ij} - minX_{ij}\right) \text{ (S1)}$$

Then, the standardized data Yij are substituted into Formula (S2) for normalization to obtain Pij.

$$P_{ij}=Y_{ij}/\sum_{m}^{i=1} Y_{ij} \; (S2)$$

The normalized data Pij are substituted into Formula (S3) to calculate the information entropy (Ej) of each indicator.

$$E_j=-1/ln(n)\sum_{i=1}^{m} P_{ij}lnP_{ij} \; (S3)$$

The information entropy is substituted into Formula (S4) to calculate the weight (ωj) of each indicator.

$$\omega_j=\left(1-E_j\right)/\sum_{i=1}^{m}\left(1-E_j\right) \; (S4)$$

S1.2.5.3 Determination of Comprehensive Weights

The weights ωj of each indicator from the EWM and the weights νj of each indicator from the AHP are substituted into Formula (S5) to calculate the comprehensive weight coefficient (Wj).

$$W_j=\nu_i\times\omega_i/\sum_{i=1}^{n} \nu_i \times \omega_i (S5)$$

Then, the comprehensive weights Wj are substituted into Formula (S6) to calculate the comprehensive score (OD).

$$OD = 100 \times \sum_{i=1}^{n} W_j \times X_i/maxX_i \text{(S6)}$$

S1.2.5.4 Single Factor Experiment

First, 400 g of *G. elata* samples of similar size were taken and divided into five portions. After being soaked in alcohol, they were sliced into thin pieces. According to the single factor experimental design, the slices were processed under varying conditions: alcohol by volume (12%, 14%, 16%, 18%, 20%), drying temperature (45°C, 60°C, 75°C, 90°C, 105°C), and drying time (12 h, 18 h, 24 h, 30 h, 36 h). Three parallel trials were performed for each condition. The average contents of the target constituents were measured and calculated, appearance evaluations were conducted, comprehensive scores were computed, and the results were compared.

S1.2.5.5 Box-Behnken Design-Response Surface Method

Based on the results of the single factor experiments, the BBD-RSM was adopted. Three factors involved in the processing of *G. elata* samples were selected for investigation, namely: (A) alcohol by volume (16%, 18%, 20%); (B) drying temperature (60°C, 75°C, 90°C); and (C) drying time (24 h, 30 h, 36 h). The experimental data were analyzed using a quadratic multiple regression equation and analysis of variance via Design-Expert 8.0 software, so as to more accurately predict the optimal combination of process parameters.

S1.2.5.6 *Validation of Optimal Process and Comparison of Active Component Contents*

According to the optimization results of the response surface model, the same batch of *G. elata* materials was divided into three equal parts and processed into YPGE decoction pieces following the best optimized processing technology. According to the content determination methods described in 2.2.4 above, the contents of GAS, 4-HBA, GEP, parishins (PE, PB, PC, PA) in both GE and YPGE were determined. The comprehensive scores of the three parallel experiments of YPGE were calculated using the established scoring criteria to determine whether they were close to the predicted values and whether the optimized best processing technology for YPGE was scientifically feasible. Meanwhile, a comparative analysis of the contents in GE and YPGE was conducted to explore the effects of processing with yellow rice wine on the changes in the chemical component contents of *G. elata*.

S1.2.6 In Vitro Antioxidant Activity

S1.2.6.1 Sample Preparation

Precisely weigh 1 g of *G. elata* powder, place it in a 100 mL volumetric flask, add a mixed solution (acetonitrile: water, 3:97) to the mark, and sonicate it at 300 W and 25 Hz for 30 min. Filter the solution to obtain the GE mother liquor at a concentration of 10 mg/mL. Prepare sample solutions of different concentrations according to the reaction requirements. The processed product solutions are prepared in the same manner.

Precisely weigh 1 g of L-ascorbic acid, place it in a 100 mL volumetric flask, and add pure water to the mark to obtain the vitamin C (VC) mother liquor with a concentration of 10 mg/mL. Prepare VC solutions of different concentrations according to the reaction requirements.

S1.2.6.2 DPPH Radical Scavenging Activity

The DPPH radical scavenging capacity was measured following the method described in the literature[10], with minor modifications. Briefly, 150 μL of 0.3 mmol/L DPPH in ethanol was mixed with 50 μL of sample (GE or YPGE) at 2.0-10.0 mg/mL. After incubation in the dark (30 min, room temperature), the absorbance at 517 nm (A0) was recorded. Blank absorbance (A1) was obtained by replacing DPPH with ethanol, whereas control absorbance (A2) was measured with solvent (acetonitrile: water, 3:97, v/v) instead of sample. VC served as the positive control. The scavenging rate (%) was calculated using the following equation:

$$\text{DPPH radical scavenging rate (\%)} = [1-(A0-A1)/A2] \times 100\% \quad (S7)$$

S1.2.6.3 ABTS Radical Scavenging Activity

The ABTS radical scavenging capacity was measured following the method described in the literature[11], with minor modifications. Briefly, 150 μL of ABTS working solution was mixed with 50 μL of sample solutions (GE or YPGE) at different concentrations (2.0, 4.0, 6.0, 8.0, and 10.0 mg/mL). After incubation in the dark for 30 min, the absorbance (A0) was measured at 734 nm. For the blank group, the absorbance (A1) was determined by replacing ABTS solution with an equal volume of absolute ethanol. For the control group, the absorbance (A2) was measured by substituting the sample solution with an equal volume of the mixed solvent (acetonitrile: water, 3:97, v/v). VC served as the positive control. The ABTS radical scavenging rate (%) was calculated using the following formula:

$$\text{ABTS radical scavenging rate (\%)} = [1-(A0-A1)/A2] \times 100\% \quad (S8)$$

S1.2.6.4 Hydroxyl Radical Scavenging Activity

The hydroxyl radical scavenging capacity was measured following the method described in the literature[7], with minor modifications. Briefly, sample solutions (GE or YPGE) at various concentrations (2.0, 4.0, 6.0, 8.0, and 10.0 mg/mL) were mixed with 50 μL of $FeSO_4$ solution and 50 μL of H_2O_2 solution. After incubation for 10 min, 50 μL of salicylic acid-ethanol solution was added, and the mixture was incubated in the dark at 37℃ for 40 min. The absorbance (A0) was then measured at 510 nm. For the blank control, H_2O_2 solution was replaced with an equal volume of distilled water (A1). For the negative control, the sample solution was substituted with an equal volume of the mixed solvent (acetonitrile: water, 3:97, v/v) (A2). VC served as the positive control. The hydroxyl radical scavenging rate (%) was calculated using the following formula:

$$\text{Hydroxyl radical scavenging rate (\%)} = [1-(A0-A1)/A2] \times 100\% \quad (S9)$$

S1.2.7 Behavioral observation

After model establishment, the experimental animals were subjected to a 2-day water maze adaptation training[12], to prevent the influence of stress in rats on the experimental results during the formal experiment. After filling the pool with water, it is dyed white, and picric acid fluorescent agent is applied to the heads of rat[13], to enhance the imaging contrast.

S1.2.7.1 Positioning navigation

In the pool, four quadrants are sequentially set in the four directions of east, south, west and north, and the escape platform is placed 2 cm below the liquid surface, the water temperature was controlled at (20±2) °C, and the laboratory lighting was kept stable. The rats were allowed to enter the pool randomly from different quadrants and swim freely until they found the escape platform beneath the water surface.

S1.2.7.2 Space search

After the adaptation training is completed, place the escape platform in the fourth quadrant and gently release the rats into the pool from the second quadrant. Record the time taken for the rats to find the escape platform[14], namely the escape latency. The duration is valid within 120 seconds. If the rat fails to find the escape platform within 120 seconds, the experimental data for that trial is invalid.

S1.2.8 Detection of Inflammatory Cytokines, Oxidative Stress Indicators, and Neurotransmitters

Collection of Rat Brain Tissue: After anesthesia, cut the skin and flesh from the top of the rat's skull to the neck, after the skull is fully exposed, decapitation is used to quickly euthanize the rats, use ophthalmic scissors to cut through the entire skull from the end of the rat's head forward, apply force toward the top of the skull while cutting to avoid damaging the brain tissue. Use fine forceps to carefully lift the brain tissue from the medulla oblongata end, after cutting the connections of other blood vessels, immediately immerse in pre-cooled physiological saline for rinsing, two brain tissues are randomly selected from each group, part of the tissues are fixed with 4% paraformaldehyde solution, and the other part is rapidly frozen in liquid nitrogen, then store in a-80°C refrigerator for future use.

S1.2.8.1The Effect of YPGE on the Antioxidant Capacity of Rat Brain Tissue

SOD converts superoxide anion radicals into hydrogen peroxide, GSH-Px catalyzes the

reduction of hydrogen peroxide to water[15], they work synergistically to scavenge free radicals in the body, reduce oxidative stress injury, protect neurons, MDA is an indicator of membrane peroxidation.

S1.2.8.2 The Effect of YPGE on Neurotransmitters in Rats

Glu is an excitatory neurotransmitter[16], an increase in its content leads to excessive neuronal excitation, increase the possibility of epileptic seizures. The results of the study showed that, compared with the model group, both *G. elata* and YPGEs could significantly reduce the content of Electability, it indicates that both *G. elata* and YPGE can inhibit neuronal excitability, thus playing an anti-epileptic role.

GABA is an inhibitory neurotransmitter, can inhibit neuronal excitability, the GABA level in the model group was lower than that in the control group.

S1.2.9 The Effect of YPGE on the Hippocampus of Rat Brain Tissue

Take paraffin sections. After routine dewaxing and rehydration, immerse them in citric acid for 8 minutes, then wash with PBS buffer three times, 5 minutes each time; Incubate in TUNEL fluorescent incubation solution at 37°C for 1 hour; Then wash with PBS buffer solution three times, 5 minutes each time; Add DAPI and then mount the slides for fixation, Observe the apoptosis levels of hippocampal and cortical cells in each group of rats under a fluorescence microscope (×400), and calculate the apoptosis rate of brain neurons based on the counting results.

S1.2.10 Western Blot Analysis

Take an appropriate amount of brain tissue and rinse it with pre-cooled PBS[17], add 3 times the volume of lysis buffer(RIPA:PMSF=1000:1), homogenization, stand still in an ice bath for half an hour, after centrifugation for 10 minutes, detect the protein content in the supernatant using the BCA method, add loading buffer to the protein sample, heat in boiling water for 10 minutes[18] cool to room temperature and then freeze immediately for future use. The protein is subjected to SDS-PAGE electrophoresis, remove the film. After membrane transfer, add blocking solution and incubate overnight at 4°C, incubate the blocked PVDF membrane with primary antibody and secondary antibody respectively, and then perform development. Detect the changes in Bcl-2, Bax protein expression in rats.

References

1. Zhao, X.; Fang, W.; Liu, S.; Tian, Y.; Chen, S.; Zou, J.; et al. Study on Optimization of Extraction Technology of Gastrodia elata Polysaccharides from Xuefeng Mountain in Hu'nan. *RCCSE*. **2020**, *2*, 46-50. https://doi.org/10.16693/j.cnki.1671-9646(X).2020.01.048.
2. Hu, D.; Liu, J.; Li, F.; Ding, P.; Wang, Y.; Tian, M.; et al. Determination and optimization of the polysaccharide content of *Gastrodia Elata Bl*.f.glauca by anthrone-sulfuric acid methods. *Yunnan Minzu Daxue Xuebao, Ziran Kexueban.* **2023**, *32*, 687-694. (In Chinese)

3. Yang, L.; Qin, S.-H.; Zi, C.-T. Research progress of *Gastrodia elata* Blume polysaccharides: A review of chemical structures and biological activities. *Front. Chem.* **2024**, *12*, 1395222. https://doi.org/10.3389/fchem.2024.1395222.
4. Fu, Y.; Xu, Q.; Zhang, J.; Kang, C.; Yang, C.; Guo, L.; et al. Identifying the quality markers and optimizing the processing of *gastrodiae rhizoma* to treat brain diseases. *Front. Pharmacol.* **2024**, *15*, 1396825. https://doi.org/10.3389/fphar.2024.1396825.
5. Chen, F.; Zhang, X.; Zhang, Y.; Sun, J.; Zhang, W.; Bai, H.; et al. Study on fresh-c ut processing of *Gastrodiae Rhizoma* based on AHP-CRITIC method combined with response surface method. *Zhongcaoyao*. **2024**, *55*, 4338-4349. (In Chinese)
6. Yang, X.; Li, L.; Yan, Y.; Hu, X.; Li, Q.; Li, L.; et al. Investigation of the pharma codynamic components of *gastrodia elata blume* for treatment of type 2 diabetes m ellitus through HPLC, bioactivity, network pharmacology and molecular docking. *Int J Mol Sci*. **2024**, *25*, 10498. https://doi.org/10.3390/ijms251910498.
7. Nie, Y.; Yao, W. A comprehensive quality evaluation method based on C30-HPL C and an analytic hierarchy process for the Chinese herbal formula, erzhiwan. *M olecules*. **2018**; *23*, 2045. https://doi.org/10.3390/molecules23082045.
8. Wang, S.; Yu, S.; Yang, X.; Cui, D.; Fu, X.; Wang, Q.; et al. Evaluation of quality attributes of different parts of poria cocos during stress sweating process based on AHP-EWM and RSM. *IND CROP PROD***.** **2024**, *210*, 118047. https://doi.org/10.1 016/j.indcrop.2024.118047.
9. Jia, S.; Zhe, D.; Li, S.; Meng, J.; Jun, Z.; Chang, C.; et al. Grading of gastrodia el ata pieces based on quality constant. *Zhongguo Zhongyao Zazhi*. **2019**, *44*, 1750-175 4. (In Chinese)
10. Zhao, X.; Zhu, M.; Ren, X.; An, Q.; Sun, J.; Zhu, D. A new technique for determ ining micronutrient nutritional quality in fruits and vegetables based on the entr opy weight method and fuzzy recognition method. *Foods*. **2022**, *11*, 3844. https://d oi.org/10.3390/foods11233844.
11. Chen, G.; Fang, C.; Chen, X.; Wang, Z.; Wang, M.; Kan, J. High-pressure ultraso nic-assisted extraction of polysaccharides from mentha haplocalyx: Structure, func tional and biological activities. *IND CROP PROD*. **2019**, *130*, 273-284. https://doi.o rg/10.1016/j.indcrop.2018.12.086.
12. Shi, X.; Wang, L.; Zhang, S.; Zhou, Y.; Wan, Y. Chemical constituents from *Gastr odia elata* and their antioxidant and α-glucosidase inhibitory activities. *Zhongcaoya o*. **2024**, *55*, 6474-6481. (In Chinese)
13. Sunil, K.; Reddy, T.; Sekharan, C. Utility of picric acid and 2,4 dinitrophenol as chromogenic reagents for visible spectrophotometric quantification of alogliptin. *B ulletin of Faculty of Pharmacy, Cairo University*. **2017**, *55*, 177-184. https://doi.org/10. 1016/j.bfopcu.2017.02.002.
14. Marafioti, T.; Lozano, M.; Andrea, C. Characterization of the immune infiltrate in mouse tissue by multiplex immunofluorescence. *RADIOTHER ONCOL*. **2023**, *174*, 43-53. https://doi.org/10.1016/BS.MCB.2022.07.003.
15. Cheng, M.; Yuan, C.; Ju, Y.; Liu, Y.; Shi, B.; Yang, Y.; et al. Quercetin Attenuates Oxidative Stress and Apoptosis in Brain Tissue of APP/PS1 Double Transgenic AD Mice by Regulating Keap1/Nrf2/HO-1 Pathway to Improve Cognitive Impairment. *Behav Neurol*.

2024, *2024*, 5698119. https://doi.org/10.1155/2024/5698119.

16. Jing, W.; Qing, C.; Xia, Y.; Ning, Q.; Demei, X.; Xue, W.; et al. Inhibiting SNX14 Alleviates Epileptic Seizures by Regulating GluA2 Degradation via the Lysosomal Pathway. *Mol Neurobiol*. **2025**, *62*, 10902-10914. https://doi.org/10.1007/s12035-025-04945-y.
17. Lazzaro, S.; West, M.; Eatemadpour, S.; Feng, B.; Varma, MVS.; Rodrigues, AD.; et al. Translatability of in vitro Inhibition Potency to in vivo P-Glycoprotein Mediated Drug Interaction Risk. Pharm Sci. **2023**, 112, 1715-1723. https://doi.org/10.1016/j.xphs.2023.01.014.
18. He, L.; Yu, L.; Yu, G. Traditional Chinese medicine Ze-Qi-Tang formula reduces inflammation in mice with asthma by inhibiting PI3K/AKT/NF-κB signaling pathway. *CELL MOL BIOL*. **2024**, 70, 170-174. https://doi.org/10.14715/cmb/2024.70.8.24.
